# Wheat seed germination prediction in response to temperature, water potential, and salinity using an artificial neural network

**DOI:** 10.1038/s41598-026-44918-2

**Published:** 2026-04-16

**Authors:** Hedayatollah Karimzadeh Soureshjani, Mahmoud Bahador, Ayoub Ghorbani Dehkordi, Hamideh Ghaffari

**Affiliations:** 1 Department of Agronomy, Sari Agricultural Sciences and Natural Resources University (SANRU), Sari, Iran; 2 Fars Agricultural and Natural Resources Research and Education Center, Agricultural Research, Education and Extension Organization (AREEO), Shiraz, Iran; 3 Department of Horticulture, Gorgan University of Agricultural Sciences & Natural Resources (GUASNR), Gorgan, Iran; 4 State Key Laboratory for Wheat Improvement, College of Life Sciences, Shandong Agricultural University, Taian, 271018 China; 5 AgroBioTech Research Center, Slovak University of Agriculture, Nitra, Slovakia

**Keywords:** Abiotic stresses, Artificial intelligence modeling, Multi-layer perceptron, Seed germination simulation, Environmental sciences, Plant sciences

## Abstract

Multi-layer perceptron (MLP) neural networks can be used to develop accurate models for quantifying plant responses to environmental factors. This study aimed to quantify wheat seed germination in response to temperature, water potential, and salinity using an ANN. Results indicated that the MLP model could predict total germination percentage with high model accuracy, including R^2^ (0.99), MSE (0.342), RMSE (0.585), and MAE (2.166) for the test data. Time to 50% germination (T50) was also accurately estimated using the MLP model (R^2^ = 0.97, MSE = 26.2, RMSE = 5.11, MAE = 5.70). Water potential was identified as the most significant variable affecting total seed germination and T50, followed by salinity and temperature. Seed germination was maximum at 20.5 °C and decreased at higher and lower temperatures. The optimal temperature for T50 was 25.3 °C. Higher salinity and more negative water potential led to lower total seed germination. The results of this study can be used to develop process-based models of crop growth and development and predict total seed germination and germination time under different conditions of temperature, water potential, and salinity.

**Supplementary Information:**

The online version contains supplementary material available at 10.1038/s41598-026-44918-2.

## Introduction

Wheat (*Triticum aestivum* L.), the most widely cultivated crop worldwide, provides about a fifth of the total calories consumed by humans^1^. Therefore, any improvement in the reaction of wheat to environmental factors such as temperature, water potential, and salinity can help improve the production of this plant and reduce hunger in the world^2,3^. Today, the phenomenon of climate change and global warming has caused plants to be more exposed to various stress conditions, such as temperature, water, and salinity stresses^4,5^. These extreme conditions adversely affect crop growth, development, and yield, but the effects of stressors vary depending on stress severity, crop species, and growth stage.

Germination is the essential among crop growth stages because successful germination is a prerequisite for subsequent stages and the production of economic yield^6,7^. Seed germination is a biological process influenced by various environmental and genetic factors^8,9^. Environmental factors such as moisture, temperature, salinity, and light have different effects on seed germination depending on plant species and stress severity^10^. The maximum seed germination percentage occurs when all environmental factors (temperature, water potential, salinity, etc.) are optimal in a non-dormant, healthy seed lot with high seed viability. When environmental factors are outside the optimal range, seed germination declines, and the time to a given fraction of germination (e.g., 50%) is delayed^6,11^.

Quantitative knowledge of the effect of environmental factors on seed germination helps predict crop germination time and the maximum germination rate. There are different methods for quantifying seed germination response to environmental factors. There are several methods of calculating seed germination response to temperature, for example, by beta, segmented, dent-like, and binomial models^6,9,12^. Researchers used some models, including dent-like, segmented, and beta, to simulate seed germination in wheat^12,13^, sesame (*Sesamum indicum* L.), and flax (*Linum usitatissimum* L.)^6^, and chickpea (*Cicer arietinum*)^9^ to temperature. Some methods can quantify seed germination responses to two environmental factors: temperature and water potential. For instance, hydro time (HT) and hydrothermal time (HTT) models are used to simulate seed germination under different temperature and water potential treatments^14–17^. The germination response to temperature and water potential was quantified using HT and HTT models in true potato seeds (*Solanum tuberosum* L.)^10^, sesame^18^, *Melisa officinalis* L^19^., Zucchini (*Cucurbita pepo* L.)^20^, and watermelon (*Citrullus vulgaris*)^17^. The halothermal time (HaloTT) model is used to quantify seed germination in response to salinity and temperature or water potential and temperature^21^. It simulated seed germination of chicory (*Cichorium intybus* L.) under different combinations of temperature × salinity and temperature × water potential. However, when the number of environmental factors, such as temperature, water potential, and salinity, increases, it is challenging to quantify seed germination due to the complexity and inflexibility of existing equations.

Seed germination under environmental stress—such as fluctuating temperature, water potential, and salinity—is a multifactorial and inherently nonlinear process. Traditional statistical frameworks, including ANOVA and Multiple Linear Regression (MLR), often struggle to capture the complex, interactive effects of these abiotic factors^22–24^. Consequently, Artificial Neural Networks (ANNs) have emerged as a robust alternative for modeling high-dimensional biological datasets and extracting meaningful patterns from nonlinear relationships. Among these, the Multi-Layer Perceptron (MLP) architecture is particularly effective for structured agricultural data due to its brain-inspired processing capabilities. While MLP is a well-established architecture in computer science, its application in predicting crop growth stages and physiological responses is a relatively recent development^25^. For instance, Saffariha et al.^26^ demonstrated that MLP could predict *Salvia limbata* germination with significantly higher accuracy (R^2^ = 0.94) compared to MLR (R^2^ = 0.67). Building on this proven predictive performance, the present study employs the MLP model to quantify the interactive effects of multiple stresses on germination with high precision. Therefore, this study significantly enhances predictive accuracy and provides a robust methodological approach for understanding and optimizing complex biological responses in plant systems under environmental stress by employing an ANN-based modeling framework.

## Results

### Total germination

Total seed germination ranged from 1.3% to 98.7% across different combinations of temperature, water potential, and salinity. Different MLP structures were used to predict the total germination percentage in the current study (Table 1). These MLP models differed in their inactivation and training functions, as well as their layer structures. The results showed that the structure of “3–6-10-1” with the “Logsig-Tansig-Purelin” activation function and “CGB (Conjugate Gradient Backpropagation)” training function was the best MLP model to predict seed germination of wheat at the different conditions of temperature, water potential, and salinity (Table 1). This MLP model achieved the highest accuracy, with R^2^ (0.99), MSE (0.342), RMSE (0.585), and MAE (2.166) all low on the test data. This MLP model includes three input parameters (temperature, water potential, and salinity), 6 and 10 neurons in the first and second hidden layers, and one neuron in the output layer to calculate seed germination. Therefore, logarithm sigmoid and hyperbolic tangent sigmoid transfer functions in the hidden layer, along with a linear transfer function in the output layer, are the best functions to achieve the most accurate wheat seed germination.

**Table 1:** The performance measures of the MLPs for total seed germination percentage.

Row	Activation Function	Training Function	Structure	Accuracy	Training Data	Validation Data	Test Data	All Data	
								Value	SE
1	Logsig-Tansig-Purelin	LM	3–8-8-1	R^2^	0.997	0.997	0.993	0.996	0.0012
				MSE	0.013	2.642	24.770	1.937	
				RMSE	0.114	1.625	4.977	1.392	0.14
				MAE	1.204	1.490	1.823	1.339	
2	Tansig-Purelin	LM	3–8-1	R^2^	0.984	0.982	0.983	0.986	0.0019
				MSE	2.29	0.000	19.909	5.598	
				RMSE	1.51	0.022	4.462	2.366	0.198
				MAE	2.57	2.713	3.287	2.451	
3	Logsig-Tansig-Purelin	GDM	3–10-6-1	R^2^	0.997	0.996	0.993	0.996	0.0005
				MSE	0.010	0.788	27.953	6.116	
				RMSE	0.101	0.888	5.287	2.473	0.11
				MAE	1.189	1.538	1.581	1.300	
4	Tansig-Purelin	GDM	3–8-1	R^2^	0.982	0.983	0.986	0.983	0.001
				MSE	0.0005	19.909	5.598	0.687	
				RMSE	0.022	4.462	2.366	0.829	0.198
				MAE	2.713	3.287	2.451	2.760	
5	Logsig-Tansig-Purelin	RP	3–8-8-1	R^2^	0.989	0.991	0.991	0.990	0.001
				MSE	0.171	3.476	0.892	0.0001	
				RMSE	0.413	1.864	0.944	0.010	0.144
				MAE	2.224	2.310	2.061	2.212	
6	Tansig-Purelin	RP	3–6-1	R^2^	0.982	0.983	0.985	0.982	0.001
				MSE	0.002	21.513	5.629	0.842	
				RMSE	0.049	4.638	2.373	0.918	0.196
				MAE	2.735	3.258	2.547	2.785	
7	**Logsig-Tansig-Purelin**	**CGB**	**3–6-10-1**	**R** ^**2**^	**0.992**	**0.991**	**0.990**	**0.992**	**0.0009**
				**MSE**	**4.517**	**0.533**	**0.342**	**1.613**	
				**RMSE**	**2.125**	**0.730**	**0.585**	**1.270**	**0.14**
				**MAE**	**1.887**	**2.042**	**2.166**	**1.952**	
8	Tansig-Purelin	CGB	3–7-1	R^2^	0.977	0.983	0.981	0.979	0.001
				MSE	125.182	1.096	96.698	184.274	
				RMSE	11.188	1.047	9.833	13.575	0.196
				MAE	2.954	3.301	3.175	3.039	
9	Logsig-Tansig-Purelin	LM	3–10-10-1	R^2^	0.997	0.995	0.992	0.996	0.0006
				MSE	1.899	1.120	21.651	1.115	
				RMSE	1.378	1.058	4.653	1.056	0.118
				MAE	1.180	1.706	1.856	1.360	
10	Tansig-Purelin	LM	3–10-1	R^2^	0.975	0.981	0.979	0.976	0.002
				MSE	1.997	35.719	3.359	7.762	
				RMSE	1.413	5.977	1.833	2.786	0.211
				MAE	3.433	3.338	3.307	3.400	

A Scatter plot can be used to better understand the relationship between observed (target) and predicted (output) data. In Fig. 2, the relationship between observed and predicted total seed germination data is shown across the training, validation, test, and all datasets. The correlation coefficients (*R* > 0.99 for all datasets) in this figure indicate a strong association between the observed data and MLP outputs.

**Fig. 1:**
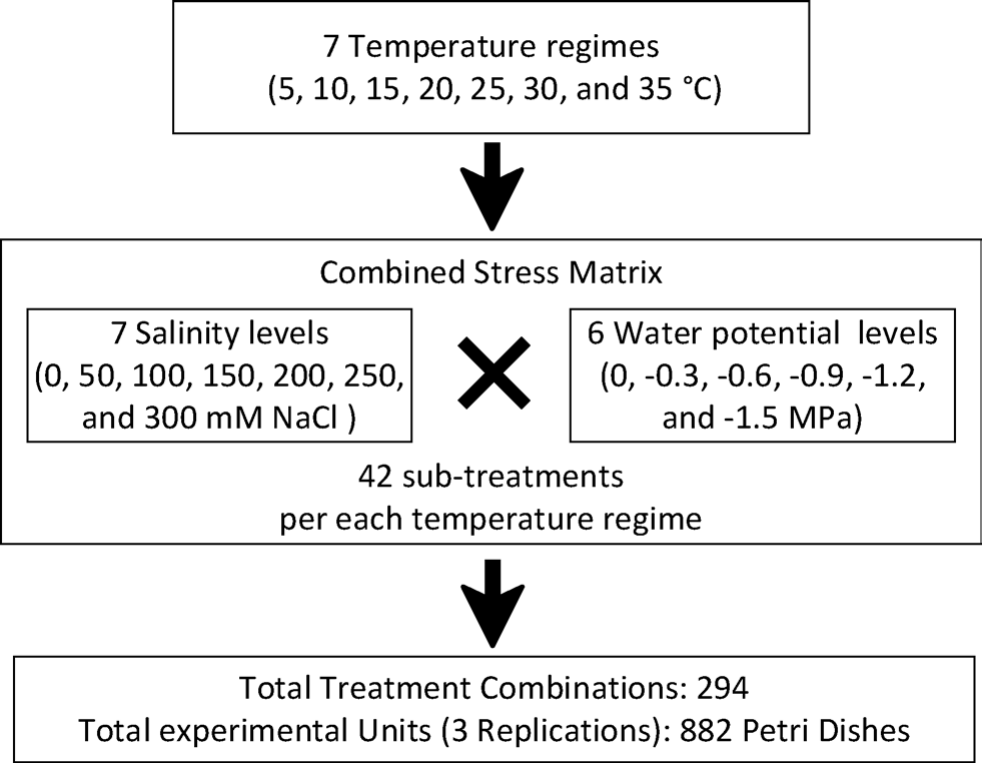
Experimental flow highlighting the nested structure of the study, where each thermal regime contained a full factorial matrix of combined salinity and osmotic potential treatments.

**Fig. 2:**
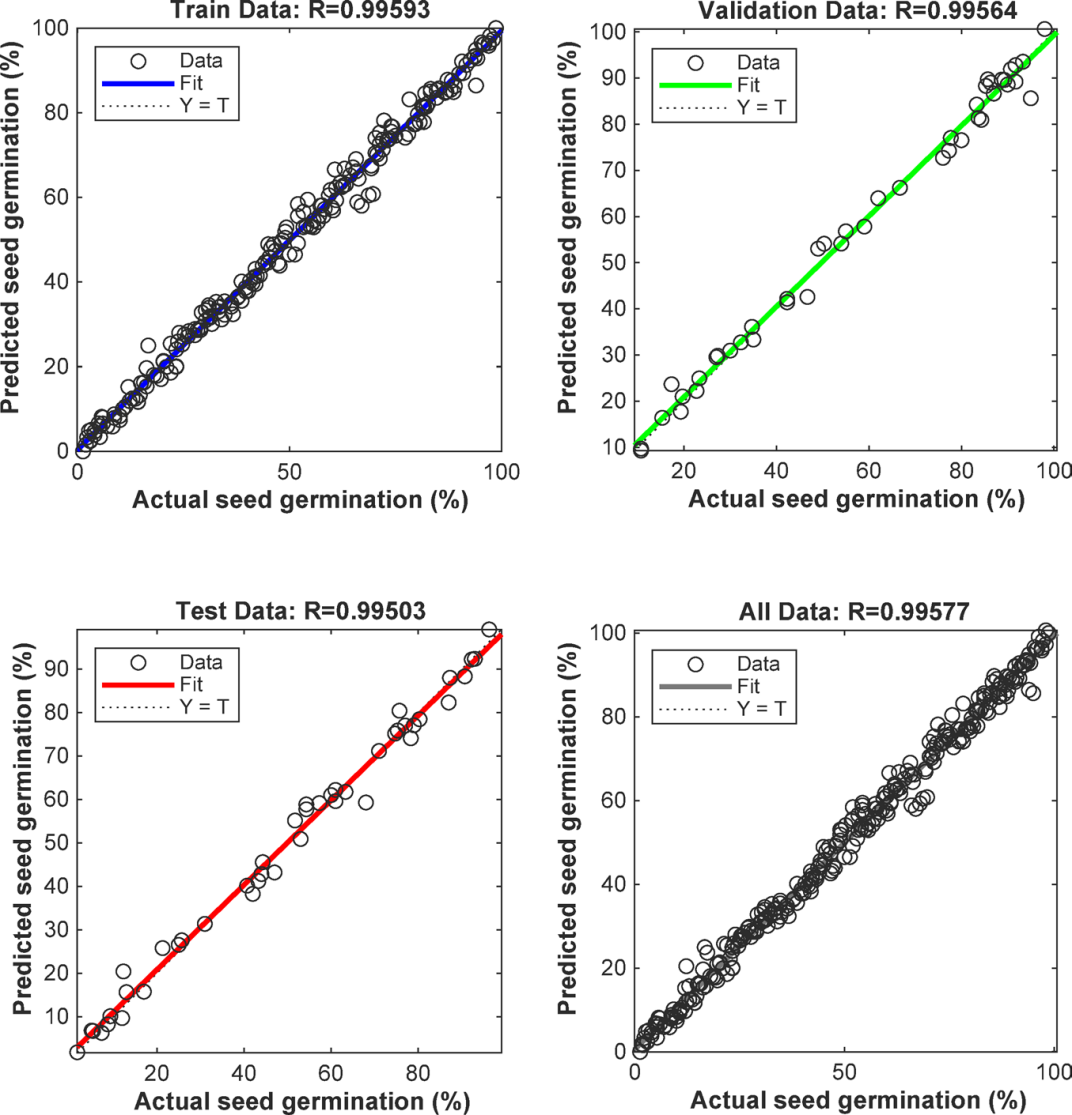
Scatter plots of output versus target total seed germination values by MLP. The solid line labeled ‘Fit’ represents the linear regression between predicted and observed values. The dashed line ‘Y = T’ denotes the 1:1 perfect agreement line, where the predicted data (Y) equals the actual data (T) value.

### T50

**Fig. 3:**
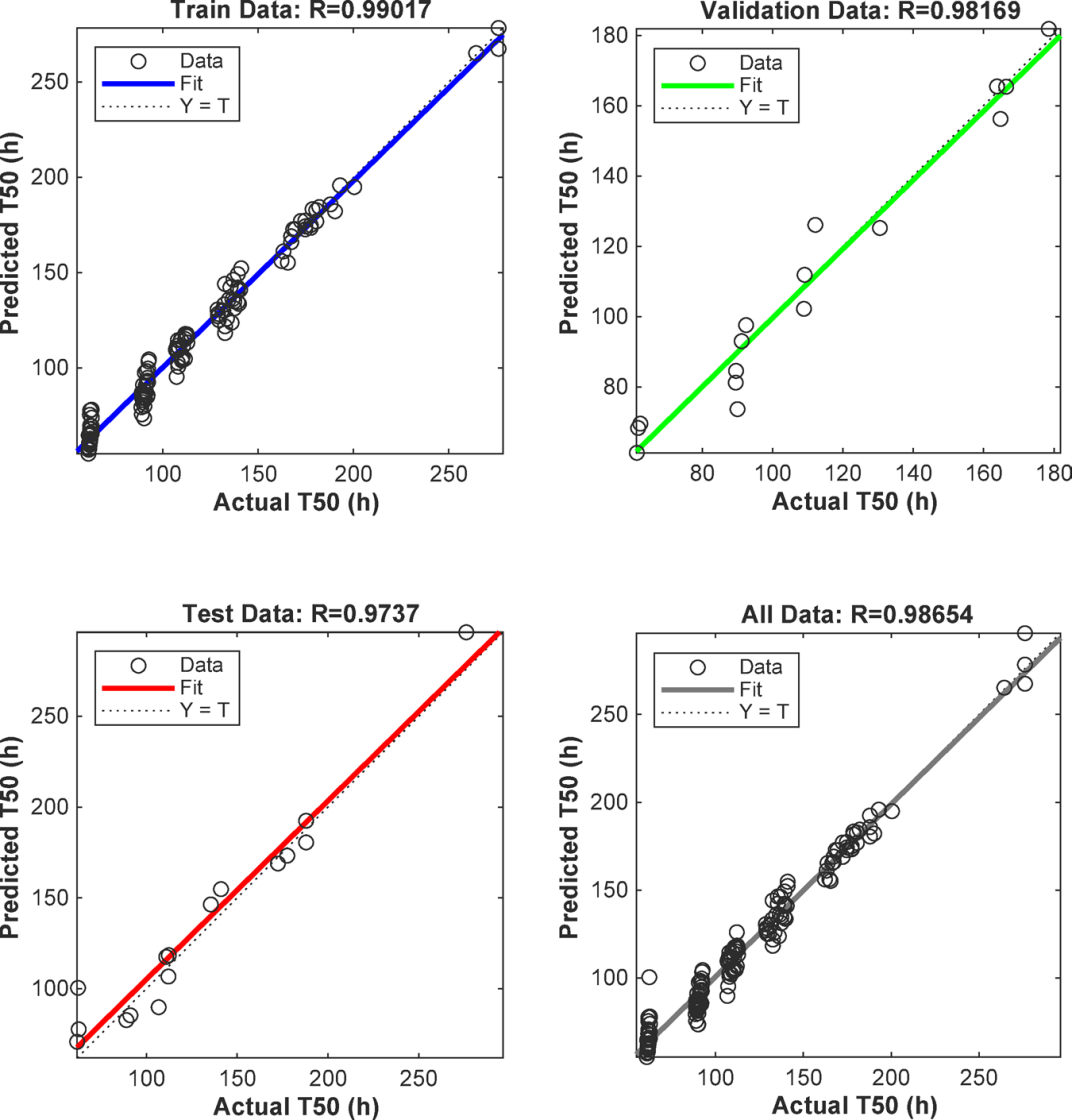
Scatter plots of output versus target time to 50% seed germination (T50) values by MLP. The solid line labeled ‘Fit’ represents the linear regression between predicted and observed values. The dashed line ‘Y = T’ denotes the 1:1 perfect agreement line, where the predicted data (Y) equals the actual data (T) value.

Only 155 of 294 treatments showed 50% germination at all combinations of temperature, water potential, and salinity. T50 of these 155 treatments were simulated using different MLPs. Based on different model accuracy parameters, the structure “3–10-6-1” with the “GDM” training function and the “Logsig-Tansig-Purelin” activation function was found to be the optimized MLP model for the best prediction of T50. This MLP model showed the highest R^2^ (0.973) and the lowest RMSE (26.208), MSE (5.119), and MAE (5.706) for all datasets (Table 2). Similar to the total seed germination MLP model, the optimized T50 MLP model includes temperature, water potential, and salinity as inputs, two hidden layers (10 and 6 neurons), and a single neural output to calculate T50. As shown in Fig. 3, the relationship between observed and predicted T50 values is linear, with a correlation coefficient greater than 0.97 across all datasets. An explicit agreement between the observed and predicted values is evident in Fig. 3.

**Table 2:** The performance measures of the MLPs for time to 50% germination (T50).

Row	Activation Function	Training Function	Structure	Accuracy	Training Data	Validation Data	Test Data	All Data	
								Value	SE
1	Logsig-Tansig-Purelin	LM	3–8-8-1	R^2^	0.864	0.890	0.479	0.842	0.006
				MSE	4488.991	201.304	4012.403	7404.809	
				RMSE	67.000	14.188	63.344	86.051	0.633
				MAE	12.115	13.241	16.363	12.912	
2	Tansig-Purelin	LM	3–8-1	R^2^	0.871	0.835	0.742	0.856	0.022
				MSE	187.354	472.676	667.520	170.456	
				RMSE	13.688	21.741	25.836	13.056	1.1
				MAE	13.902	14.936	11.552	13.706	
3	**Logsig-Tansig-Purelin**	**GDM**	**3–10-6-1**	**R** ^**2**^	**0.980**	**0.963**	**0.940**	**0.973**	**0.014**
				**MSE**	**0.027**	**5.036**	**347.068**	**26.208**	
				**RMSE**	**0.163**	**2.244**	**18.630**	**5.119**	**1.54**
				**MAE**	**5.018**	**5.856**	**10.848**	**5.706**	
4	Tansig-Purelin	GDM	3–10-1	R^2^	0.863	0.833	0.700	0.847	0.017
				MSE	474.665	578.899	774.397	388.935	
				RMSE	21.787	24.060	27.828	19.721	1.01
				MAE	14.458	15.580	12.262	14.299	
5	Logsig-Tansig-Purelin	RP	3–8-6-1	R^2^	0.801	0.632	0.583	0.758	0.028
				MSE	20.419	504.018	7.416	14.514	
				RMSE	4.519	22.450	2.723	3.810	1.56
				MAE	15.873	22.380	16.835	16.981	
6	Logsig-Tansig-Purelin	RP	3–6-1	R^2^	0.728	0.573	0.472	0.685	0.03
				MSE	0.770	1907.311	240.283	134.274	
				RMSE	0.877	43.673	15.501	11.588	1.83
				MAE	18.460	22.420	19.738	19.237	
7	Tansig-Tansig-Purelin	CGB	3–6-10-1	R^2^	0.975	0.920	0.857	0.958	0.009
				MSE	0.003	124.966	183.210	0.907	
				RMSE	0.053	11.179	13.535	0.952	0.64
				MAE	5.102	10.306	9.976	6.597	
8	Tansig -Purelin	CGB	3–7-1	R^2^	0.774	0.419	0.624	0.706	0.037
				MSE	0.003	303.684	318.865	0.014	
				RMSE	0.057	17.427	17.857	0.118	2.45
				MAE	15.990	25.985	15.123	17.345	
9	Logsig-Tansig-Purelin	LM	3–10-10-1	R^2^	0.979	0.956	0.887	0.969	0.006
				MSE	1.586	27.958	103.667	24.038	
				RMSE	1.259	5.288	10.182	4.903	0.45
				MAE	5.727	8.108	8.311	6.464	
10	Tansig-Purelin	LM	4–8-1	R^2^	0.863	0.833	0.700	0.847	0.026
				MSE	474.665	578.899	774.397	388.935	
				RMSE	21.787	24.060	27.828	19.721	1.07
				MAE	14.458	15.580	12.262	14.299	

### Sensitivity analysis of MLP

**Fig. 4:**
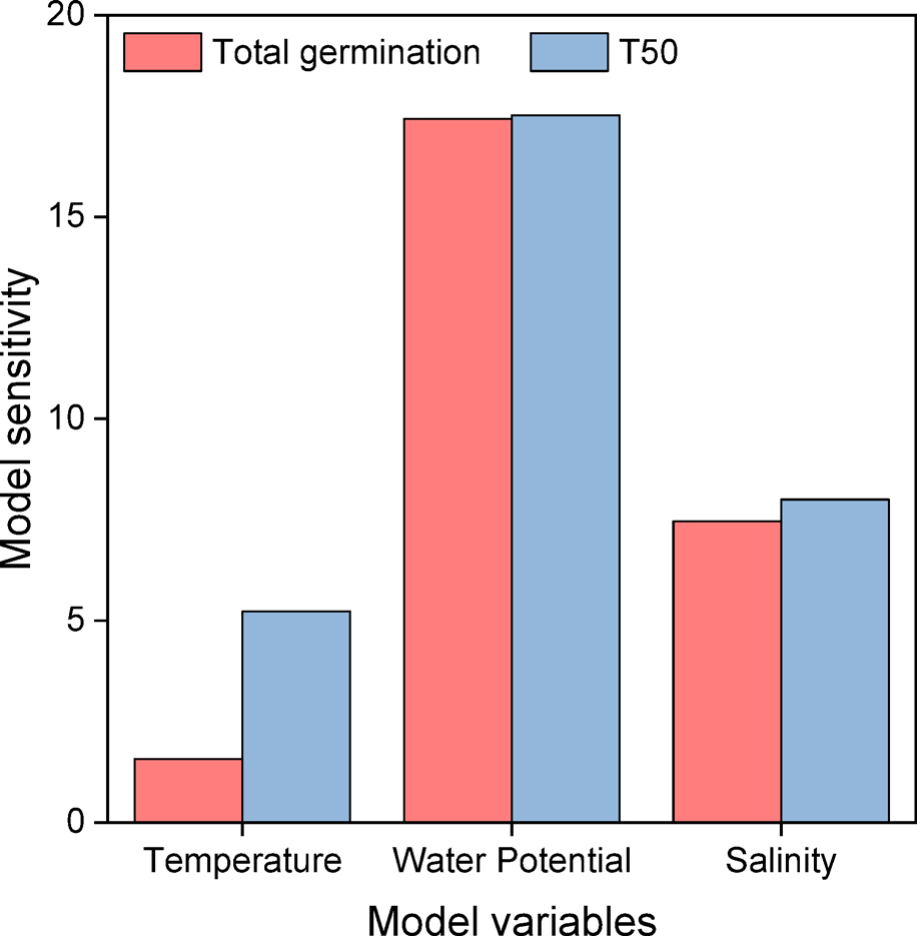
The results of sensitivity analysis of MLP for total seed germination and time to 50% seed germination (T50).

**Fig. 5:**
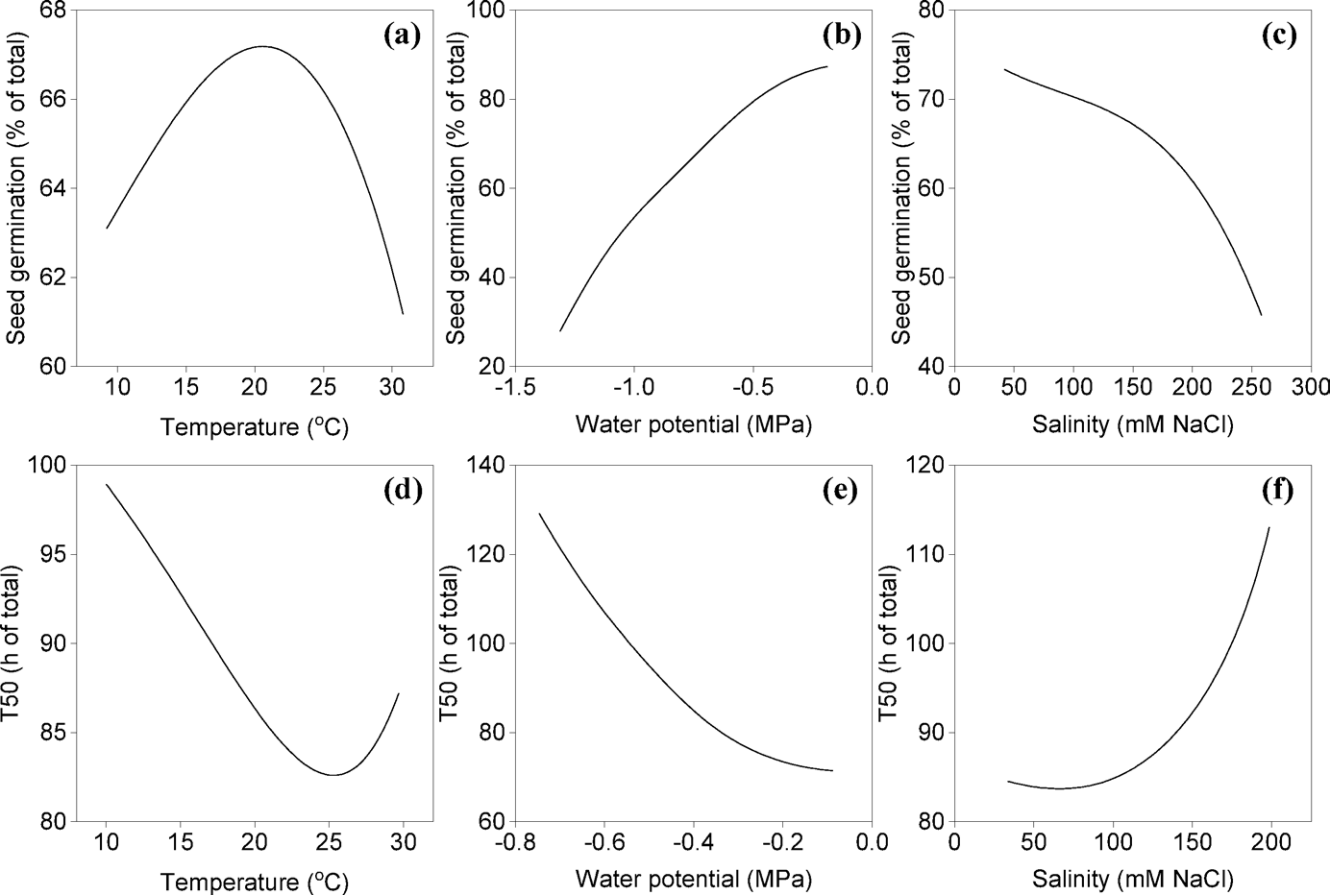
Output of sensitivity analysis of MLP for inputs variables; total seed germination percentage (**top**) temperature (**a**), water potential (**b**), salinity (**c**); time to 50% seed germination (**bottom**) temperature (**d**), water potential (**e**), salinity (**f**).

Sensitivity analyses of the most accurate MLP model were conducted for both total seed germination and T50. Results are shown in Fig. 4. Water potential, salinity, and temperature are recognized as critical variables affecting seed germination and T50. Sensitivity analysis further revealed that, among these factors, water potential exerted the greatest influence on wheat germination, followed by salinity and temperature (Fig. 4). The results showed that seed germination increased with temperature increment, reached the maximum at 20.5 °C, and then revealed a decreasing trend (Fig. 5a). Seed germination is also increased with water potential increment (more positive amount) with the highest at −0.2 MPa (Fig. 5b). Salinity had a decreasing effect on seed germination, so that seed germination was the maximum at 42 mM and started to decrease with the increase of salinity (Fig. 5c). Model sensitivity analysis also revealed that T50 has a decreasing trend with temperature increasing until it reached 25.3 °C. At the same time, a trend toward higher temperatures was observed (Fig. 5d). T50 reduced with water potential increasing (more positive amount) and raised with salinity enhancement (Fig. 5e, f). The lowest T50 was observed at a water potential of −0.1 MPa and a salinity of 67 mM.

## Discussions

In the current study, different MLP models were compared to predict wheat seed germination in response to temperature, water potential, and salinity. The other machine learning algorithms, including Random Forests and Support Vector Machines, have been applied in similar contexts. In this study, however, the Multilayer Perceptron (MLP) was selected because of its strong ability to model nonlinear relationships and its superior predictive stability^27,28^. Our findings indicated that the MLP model with a “3–6-10-1” structure, the “Logsig-Tansig-Purelin” activation function, and the “CGB” training function was the most accurate model (R^2^ = 99) for predicting seed germination. The literature contains evidence that the MLP model is more precise than other methods for predicting germination. For instance, Saffariha et al.^26^ compared the MLP model and multilinear regression (MLR) method to predict seed germination of *Salvia limbate* under different levels of temperature, salinity, water potential, and pH. They observed that the MLP model (R^2^ = 0.94) was more accurate than MLR (R^2^ = 0.67) for predicting seed germination. In the other methods of seed germination prediction, like thermal time or hydrothermal time models, ecological conditions are limited to one or two factors, including temperature and water potential^29–34^. On the other hand, the artificial neural network can predict seed germination under a broader range of environmental conditions, including temperature, salinity, water potential, pH, and planting depth^26,35^. The ANN modeling approach used in this study had the advantages of previous models^6,36,37^ and included new artificial intelligence techniques to examine the effect of more factors simultaneously on germination.

In addition to the rate of occurrence of each phenological stage in the plant life cycle, the time of its occurrence is also essential. Germination time is an essential parameter in crop operation planning and plant modeling. Knowing the time of germination would help researchers predict the next crop phenological stages, such as flowering and ripening, and schedule irrigation, fertilization, and other management practices. In the current study, an artificial neural network approach was used to predict germination time under different temperature, water potential, and salinity conditions. Results showed that the MLP model with the structure “3–10-6-1,” “GDM” training function, and “Logsig-Tansig-Purelin” activation function was the optimized MLP model for the best prediction of T50 (R^2^ = 0.94 for test data and R^2^ = 0.97 for all data). Hitherto, the time to 50% germination has been used as a criterion for seed germination stage in different crops, with various functions, such as beta, dent-like, binomial, segmented, etc^6–9,38^. In contrast to seed germination percentage, these functions (beta, dent-like, binomial, etc.) cannot quantify the time of germination as environmental factors increase. The MLP modeling approach can overcome this limitation and predict germination time with a very close approximation.

Sensitivity analysis of the MLP models for both response variables revealed that water potential was the most significant factor, followed by salinity and temperature. The results showed that seed germination was maximum at 20.5 °C and decreased at higher and lower temperatures. The optimum temperature for T50 was equal to 25.3 °C. Higher salinity and more negative water potential led to reduced total seed germination, which is similar to^26,39,40^. Lower optimum temperature for maximum germination compared with T50 reflects a well-known physiological trade-off: germination rate increased with temperature due to accelerated metabolism (Q₁₀ effect), while final germination declined beyond a thermal threshold, inducing oxidative or membrane stress and partial thermo-inhibition^41,42^. Thus, higher temperature (25.3 °C) promotes faster but less complete germination, whereas moderate temperature (20.5 °C) ensures maximum germinability through greater cellular stability.

While this study provides a robust model for predicting germination under varying water, salinity, and temperature levels, it is important to acknowledge certain environmental constraints. Other ecological factors, such as light quality and gaseous composition (e.g., oxygen and carbon dioxide concentrations), were maintained at constant, non-limiting levels during the experiments. Given that these variables can significantly modulate seed physiological responses under stress, their interactive effects with abiotic factors remain a critical area for future investigation. Incorporating these parameters into future machine learning frameworks could further enhance the predictive accuracy and ecological breadth of seed germination models.

## Conclusion

In the current study, the effects of different combinations of temperature, water potential, and salinity on seed germination of various wheat varieties were examined using an artificial neural network approach. The MLP model could accurately predict total seed germination and T50. The value of the simultaneous multi-factorial approach used in this study becomes evident when compared to established single-factor methodologies. The primary improvement of this method is its ability to reveal the non-linear interaction between salt ions and osmotic potential at varying temperatures. Our findings reveal that the inhibitory effect of NaCl is significantly amplified by decreasing water potential—a synergistic response that cannot be fully captured through isolated stress assays. This benchmarking demonstrates that our model avoids the ‘overestimation of tolerance’ common in standard protocols, which is critical for developing more robust germination models for field-scale applications where multiple abiotic stressors coexist and dynamically interact. The germination predictions generated by our model can be directly integrated into crop growth modeling frameworks. The optimal germination conditions and variable response thresholds identified here provided parameter values that can be incorporated into future process-based germination models. Since early seedling emergence strongly influences subsequent biomass accumulation, canopy development, and yield formation, accurate estimation of germination timing provides a more realistic starting point for dynamic crop simulation models. These models can improve the precision of growth trajectories across diverse environmental scenarios by incorporating MLP-based germination outputs as initial conditions, thereby enhancing management decisions on planting dates, cultivar selection, and irrigation scheduling.

“ fully captured through isolated stress assays. **This benchmarking demonstrates that our model avoids the ‘overestimation of tolerance’ common in standard protocols**, which is critical for developing more robust germination models for field-scale applications…”.

## Materials and methods

### Seed germination test

#### Treatments

The experiment was conducted as a tri-factorial arrangement within a completely randomized design (CRD) with three replications at Shahrekord University, Shahrekord, Iran. The treatments comprised seven temperature regimes (5, 10, 15, 20, 25, 30, and 35 °C), seven salinity levels (0, 50, 100, 150, 200, 250, and 300 mM NaCl), and six water potentials (0, −0.3, −0.6, −0.9, −1.2, and − 1.5 MPa), totaling 294 treatment combinations. To simulate field conditions where drought and salinity often co-occur, salinity and osmotic stresses were applied simultaneously at each constant temperature (Fig. 1). This synergistic protocol established a full factorial matrix of salinity and osmotic potentials within the germinators, allowing for a systematic evaluation of the interactive effects of these abiotic stresses across a broad thermal range. NaCl solutions (Merck, Germany) were prepared at the desired concentrations, and their water potential was measured using a vapor pressure osmometer (Model 5100 C; Wescor Inc., Logan, UT, USA). The total water potential was then adjusted to the target level by adding the required amounts of polyethylene glycol (PEG 6000, Merck, Germany) based on the method of Michel and Kaufmann (1973)^43^.

Pishgam, a wheat variety suitable for cold regions, was selected for this study. The viability of seeds was measured on 50 seeds in four replicates at 20 °C^6^, which was 99.2%, and the thousand-seed weight was also 46 g.

In the germination test, 50 seeds were randomly selected from the seed lot for each Petri dish, which were first sterilized with 5% sodium hypochlorite, then placed on Whatman filter paper (Cat. No. 1001 150) in a Petri dish containing 10 mm of a combination of each water potential and salinity treatment. Seeds were then placed in the incubator (model IF260, Memmert GmbH + Co. KG, Germany) at each temperature treatment in the dark, except during brief observations for counting. To minimize evaporation and ensure stable solution concentrations, the incubator’s relative humidity was maintained at 95%. Seeds were exposed to normal ambient CO₂ levels, which are adequate for wheat germination.

Germinated seeds were counted several times a day based on temperature and the observed germination rate. The germination criterion was reaching a radicle length of two mm^6,9^. Recording of germinated seeds was stopped when no new seeds germinated for two consecutive days^6,7^. Total germinated seed (%) was calculated by dividing germinated seed by total seed in each Petri dish, multiplying by 100 (Eq. 1). A logistic model Eq. 2 was fitted to cumulative seed germination (%) versus time (h) to determine the time of 50% germination (T50). In the current study, germination occurred in only 50% of the treatments.

1 $$\:{G}_{X}=\:\frac{n}{N}\times\:100$$

2 $$\:G=\frac{{G}_{X}}{1+exp\left[a\right(t-b\left)\right]}$$

n; germinated seeds

N; total seeds

$\:{G}_{X}$; total germination percentage

G; cumulative germination percentage

t; time

b; time to 50% germination

a; parameter

### Multi‑layer perceptron neural network

MLP structure similar to the human brain uses neurons to learn relationships between inputs (temperature, water potential, and salinity) and outputs (total germination or T50)^26^. In neurons, a transfer function summarizes weighted inputs to predict the output^44^. In each neuron, the weighted input variables are connected to the variables of the other neurons. The weights of the hidden layers are continuously adjusted using a learning algorithm to minimize the difference between the target and the network output^45,46^. Model optimization was achieved through an iterative, experience-driven approach, in which the number of hidden layers and neurons was manually adjusted based on convergence stability, error minimization, and model simplicity^47^. To ensure the robustness and generalizability of the predictive models, the dataset was randomly partitioned into three subsets: training (70%), validation (15%), and testing (15%), using a fixed random seed to guarantee reproducibility. The network architecture was optimized to balance complexity and performance, preventing overfitting by avoiding excessively deep or wide configurations. Specifically, the validation set was utilized to monitor model performance and implement an early stopping criterion; training was terminated when the error on the validation set began to increase despite a continued decrease in training error (overtraining). This approach ensured that the MLP model maintained its ability to generalize to unseen data while achieving high predictive accuracy for both total seed germination and T50.

The most common activation functions, including the logistic sigmoid, hyperbolic tangent, sigmoid tangent, and linear transfer, were used in the current study to optimize MLP performance. The back propagation (BP) method is generally used in the learning process to regulate the optimal weight and bias of neurons^26,48^.

The BP method is used to reduce the difference between the predicted (MLP output) and target (total seed germination percentage, T50) by adjusting the weights of neurons (w) and input variables (x). In this regard, the weight of each variable changes in each neuron continuously during the training process. Weight adjusting of a neuron output in a hidden layer is calculated by Eq. 3^49^. Then, some transfer functions such as logarithmic sigmoid, hyperbolic tangent, sigmoid tangent, and linear are used in the structure of hidden layers, and neuron output is calculated using Eq. 4. The selection of transfer functions is based on trial and error to find the best model and reduce errors^49^.

3 $$\:{net}_{j}^{k}=\:\sum\limits_{i=0}^{n}{w}_{ji}{x}_{ji}$$

4 $$\:{Y}_{net}=\:\int\:{net}_{j}$$

i; ith variable

j; jth neuron

k; kth hidden layer

x; weight of variables

w; weight of neurons

Y; MLP output

In the current study, the weight of 294 samples for total seed germination and 155 samples for T50 was adjusted by the delta rule which has been summarized in Eq. 5. The difference between the total treatments (294) and those with calculable T₅₀ values (155) was because under extreme stress conditions, such as high salinity, low water potential, or unfavorable temperatures, germination did not reach 50%. Although excluding these treatments may introduce some bias, it accurately reflects biological reality, as germination was severely inhibited under such stresses. Therefore, the T₅₀ model was developed only for treatments with measurable germination dynamics to ensure reliable and interpretable results.

5 $$\:{w}_{ji}^{t}=\:{w}_{ji}^{t-1}+\:\left(-\:\frac{{\partial\:E}^{t}}{{\gamma\:\partial\:w}_{ji}^{t}}\right)$$

E; the sum of squared errors between predicted and target valueswji; represents the weight of the ith neuronin the jth hidden layer$\:\gamma\:$; learning ratet; target output.

### Analysis and model accuracy assessment

Logistic model fitting was performed in the SAS 9.4 environment using the NLIN procedure. The artificial neural network was also run in MATLAB 2018b. For this purpose, temperature, water potential, and salinity were input, and the total seed germination percentage (T50) was output. Model accuracy estimation was performed using the coefficient of determination (R^2^, Eq. 6), mean squared error (MSE, Eq. 7), root mean square error (RMSE, Eq. 8), and mean absolute error (MAE, Eq. 9)^6,50,51^. The MLP model with a higher R^2^ and lower MSE, RMSE, and MAE is more accurate.

6 $$\:{R}^{2}=\frac{SSR}{SST}$$

7 $$\:MSE=\:\frac{\sum\:_{i=1}^{n}{({O}_{i}-\:{P}_{i})}^{2}}{n}$$

8 $$\:RMSE=\sqrt{\frac{1}{n}{\sum\:}_{i=1}^{n}{({O}_{i}-{P}_{i})}^{2}}$$

9 $$\:MAE=\frac{1}{n}\:\sum\:_{i=1}^{n}\left|{O}_{i}-{P}_{i}\right|$$

n; number of samples

$$\:{O}_{i}$$; observed values

$$\:{P}_{i}$$; predicted values

$\:{O}_{ave}$; mean measured value

$\:{P}_{ave}$; the average of the predicted value

SSR: sum of squares of regression

SST: total sum of squares

The importance of each variable, including temperature, water potential, and salinity, was determined through a sensitivity analysis of the MLP model on its output. Thus, the standard deviation was calculated for each variable, and each variable was shifted within the range of standard deviation (mean ± SD) to identify changes in model outputs. While a variable is varied within its standard deviation, the other variables are fixed at their mean values to determine the model’s sensitivity to the target variable alone. The standard deviation of the outputs in response to changes in each variable was used as the sensitivity value for that variable. Although this method does not explicitly capture potential interaction effects among environmental factors, it provides a clear and direct interpretation of the individual contribution of each variable to the studied trait response. This approach has been widely applied in ecological and environmental modeling because of its simplicity, transparency, and substantial explanatory value in identifying key drivers of system behavior^26,44^.

## Supplementary Information

Below is the link to the electronic supplementary material.

Supplementary Material 1

Supplementary Material 2

## Data Availability

All data generated or analyzed during this study are included in this published article and its supplementary information files. The datasets analyzed during the current study available from the corresponding author on reasonable request.

## Abbreviations

ANN: Artificial neural network
MLP: Multi-Layer Perceptron
T50: Time to 50% germination
