# Supplementary figures and images for: Wheat seed germination prediction in response to temperature, water potential, and salinity using an artificial neural network
Source: Sci Rep. 2026 Apr 16;16:17732. doi: 10.1038/s41598-026-44918-2 (PMC13246773; doi:10.1038/s41598-026-44918-2)

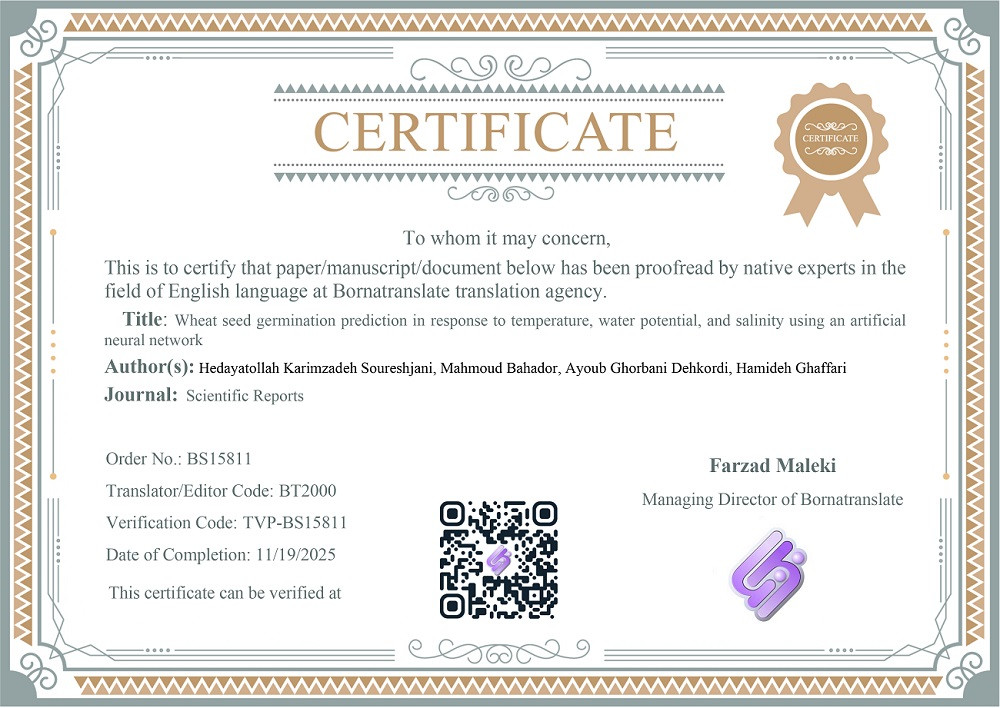

Supplement: Supplementary file 2 — Supplementary Material 2 [file 41598_2026_44918_MOESM2_ESM.jpg]
